# Midbrain microglia mediate a specific immunosuppressive response under inflammatory conditions

**DOI:** 10.1186/s12974-019-1628-8

**Published:** 2019-11-22

**Authors:** Miguel Angel Abellanas, Marta Zamarbide, Leyre Basurco, Esther Luquin, Marta Garcia-Granero, Pedro Clavero, Patxi San Martin-Uriz, Amaia Vilas, Elisa Mengual, Sandra Hervas-Stubbs, Maria S. Aymerich

**Affiliations:** 1 Departamento de Bioquímica y Genética, Universidad de Navarra, Facultad de Ciencias, Pamplona, Spain; 2 Universidad de Navarra, CIMA, Programa de Neurociencias, Pamplona, Spain; 3 Departamento de Patología, Anatomía y Fisiología, Universidad de Navarra, Facultad de Medicina, Pamplona, Spain; 4 Servicio de Neurología, Complejo Hospitalario de Navarra, Pamplona, Spain; 5 Universidad de Navarra, CIMA, Programa de Oncohematología, Pamplona, Spain; 6 Universidad de Navarra, CIMA, Programa de Inmunología, Pamplona, Spain; 7 IdiSNA, Instituto de Investigación Sanitaria de Navarra, Pamplona, Spain

**Keywords:** Microglia, Midbrain, Antigen-presenting cells, Innate immunity, TGFβ, MHC-II, Treg, LPS

## Abstract

**Background:**

Inflammation is a critical process for the progression of neuronal death in neurodegenerative disorders. Microglia play a central role in neuroinflammation and may affect neuron vulnerability. Next generation sequencing has shown the molecular heterogeneity of microglial cells; however, the variability in their response to pathological inputs remains unknown.

**Methods:**

To determine the effect of an inflammatory stimulus on microglial cells, lipopolysaccharide (LPS) was administered peripherally to mice and the inflammatory status of the cortex, hippocampus, midbrain, and striatum was assessed. Microglial activation and interaction with the immune system were analyzed in single cell suspensions obtained from the different brain regions by fluorescence-activated cell sorting, next generation RNA sequencing, real-time PCR, and immunohistochemical techniques. Antigen-presenting properties of microglia were evaluated by the ability of isolated cells to induce a clonal expansion of CD4^+^ T cells purified from OT-II transgenic mice.

**Results:**

Under steady-state conditions, the midbrain presented a high immune-alert state characterized by the presence of two unique microglial subpopulations, one expressing the major histocompatibility complex class II (MHC-II) and acting as antigen-presenting cells and another expressing the toll-like receptor 4 (TLR4), and by the presence of a higher proportion of infiltrating CD4^+^ T cells. This state was not detected in the cortex, hippocampus, or striatum. Systemic LPS administration induced a general increase in classic pro-inflammatory cytokines, in co-inhibitory programmed death ligand 1 (PD-L1), and in cytotoxic T lymphocyte antigen 4 (CTLA-4) receptors, as well as a decrease in infiltrating effector T cells in all brain regions. Interestingly, a specific immune-suppressive response was observed in the midbrain which was characterized by the downregulation of MHC-II microglial expression, the upregulation of the anti-inflammatory cytokines IL10 and TGFβ, and the increase in infiltrating regulatory T cells.

**Conclusions:**

These data show that the midbrain presents a high immune-alert state under steady-state conditions that elicits a specific immune-suppressive response when exposed to an inflammatory stimulus. This specific inflammatory tone and response may have an impact in neuronal viability.

## Background

Microglia are specialized populations of resident macrophages found in the central nervous system (CNS) [1], and they play a critical role in brain development and homeostasis. Despite similarities with other tissue-resident macrophages, microglia have two unique properties: their restricted prenatal origin and their longevity [2]. They are also cells that become active in injury and disease [2], and when they detect that cerebral homeostasis is altered, they shift their basal activity as surveying cells [1]. Homeostasis can be disturbed directly through the activation of different receptor types, like toll-like receptors (TLRs), or by factors associated to infection or neuronal death [3–5]. Alternatively, it can be altered indirectly through the disruption of receptor-ligand pairs like CX_3_CR1-CX_3_CL1, possibly reflecting a loss of neuronal integrity [6, 7]. Microglial activators induce changes that affect cell shape, their migration to the site of pathology, the phagocytosis of cells and debris, and the production of cytokines and chemokines that are necessary to stimulate microglia or other brain/immune cells [7]. Consequently, microglial activation produces a phenotypic diversity that drives versatile, stimulus-dependent responses [8, 9].

There is growing evidence of the diversity among microglia [1, 10, 11], and indeed, high-throughput single-cell transcriptomics has identified unique microglial subpopulations that were particularly diverse during early development. These populations were less heterogeneous in adulthood, at least until they were perturbed by damage or aging [12]. Different classes of activated microglia, disease-associated microglia, and injury-responsive microglia share a common transcriptional signature, but also, they express a number of unique transcripts that suggests they may respond to pathological conditions in different ways [12, 13]. Moreover, subtypes of microglia from the brain of patients with multiple sclerosis were phenotypically similar to subtypes of microglia in a mouse model of demyelination [12, 13], indicating that there are common features of microglial activation in these two species. Transcriptional networks that control bioenergetics and immunoregulation are the main contributors to microglial heterogeneity in adult mice [14]. Immunophenotypic variations indicate that microglia display distinct immune alertness depending on their location, suggesting that cerebellar and hippocampal microglia are in a more immune-vigilant state than their cortical and striatal counterparts [14]. However, further research is necessary to understand the functional relevance of microglial heterogeneity under physiological and pathological conditions.

There is limited information regarding the immunophenotype of midbrain microglia [15]. Genes associated with the immune response are conserved in the midbrain microglia of the substantia nigra (SN) and ventral tegmental area (VTA) [15], yet they have to be compared with striatal microglia. The midbrain contains the cell bodies and the striatum the terminals of dopaminergic neurons, and hence, they are possibly targets for strategies aimed at preventing the neurodegeneration that takes place in Parkinson’s disease (PD). Characterization of these cell subpopulations and their functional role in brain homeostasis is crucial to understand how they contribute to neuronal vulnerability in a specific disease context. We hypothesized that steady-state differences in immune surveillance could predispose a region to immune stimulation. Thus, in this study, an inflammatory response was induced in mice through the systemic administration of lipopolysaccharide (LPS), and microglial activation was studied in brain areas of susceptibility for different neurodegenerative diseases, such as the hippocampus, cortex, striatum, and midbrain. Our results show that under steady-state conditions, midbrain microglia present a more activated phenotype than their counterparts in other regions. Furthermore, we identified two unique microglia subpopulations in the midbrain, one expressing MHC-II with antigen-presenting properties and another expressing TLR4. Inflammatory conditions prompted an immune-suppressive response in the midbrain, probably to counterbalance the inflammatory reaction. In conclusion, for the first time, we define the immune state of the midbrain and its specific response to a general inflammatory reaction caused by peripheral administration of LPS.

## Materials and methods

### Animals and treatments

Adult male 3-month-old C57BL/6 J mice (27–30 g) were obtained from Envigo (Barcelona, Spain), while Tg (TcraTcrb)425Cbn (OT-II) mice were obtained from the Jackson Laboratory (Bar Harbor, ME, USA). The mice were housed at 21 °C in a humidity controlled environment on a 12-h light/dark cycle, fed ad libitum with standard rodent pellet diet (Envigo, Barcelona, Spain) and free access to water. Wild type animals received one intraperitoneal administration of LPS (O111:B4; 5 mg/kg in saline; Sigma-Aldrich, St. Louis, MO, USA) or saline alone, and they were sacrificed 48 h later. All procedures involving animals were carried out in accordance with the Spanish National Research Council’s guide for the care and use of laboratory animals, and the experimental design was approved by the Ethical Committee for Animal Testing at the University of Navarra (ref. 109-18).

### Cell suspensions

Mice were anesthetized with ketamine/xylazine and perfused transcardially with ice-cold phosphate-buffered saline (PBS). The brain regions of interest were dissected out on ice and digested at 37 °C with rotation for 30 min with papain (2 mg/mL, Worthington, Lakewood, NJ, USA) or for 15 min with collagenase D (400 units/mL, Roche, Mannheim, Germany) in Dulbecco’s PBS (Lonza, Basel, Switzerland), each containing 50 μg/mL of DNase I (Sigma-Aldrich). The tissue was then mechanically dissociated with a glass Pasteur pipette, filtered through a 70-μm nylon cell strainer, and centrifuged at 300*g* for 15 min. A 25% Percoll column was used to remove cell debris and myelin, centrifuging at 1000*g* for 10 min.

### Flow cytometry analysis

A cell suspension was prepared for each region of interest (cortex, midbrain, striatum, and hippocampus), and the cells were incubated for 5 min at room temperature with Zombie NIR Dye (BioLegend, San Diego, CA, USA) to assess their viability. The Zombie NIR Dye was quenched, and cells were washed with cytometry buffer (0.5% bovine serum albumin, 5 mM EDTA in PBS) prior to labeling the cells with different panels of fluorescent antibodies (Table 1) and incubating them for 15 min at 4 °C with the FcR blocking reagent (1:50, Miltenyi Biotec, Bergisch Gladbach, Germany). For intracellular staining of T lymphocytes, the cells were fixed and permeabilized with the Foxp3 transcription factor buffer set (Invitrogen, Carlsbad, CA, USA), and then incubated for 15 min at 4 °C with the primary antibodies (Table 1). The samples were washed with cytometry buffer and analyzed on a BD FACSCanto II flow cytometer using the BD FACSDiva Software v6.1.3 (BD Biosciences, Franklin Lakes, NJ, USA) and FlowJo 9.3 (FlowJo, Ashland, OR, USA). Microglial cells were defined as CD45^low^/CD11b^+^ and T lymphocytes as CD45^hi^/CD11b^−^/CD3^+^. Fluorescence minus one (FMO) and isotype control antibodies were used as negative controls for each marker (Additional file 1: Figure S1).

**Table 1:** Primary antibodies used for flow cytometry analysis

Antigen	Fluorophore	Clone	Dilution	Trademark
CD3ε	PerCP-Vio770	145-2C11	1:50	Miltenyi Biotec
CD4	FITC	GK1.5	1:1000	BioLegend
CD8a	PE-Cy7	53.6-7	1:1000	BioLegend
CD11b	VioBlue	M1/70.15.11.5	1:100	Miltenyi Biotec
CD25	APC	PC61	1:200	Biolegend
CD28	APC	37.51	1:50	Biolegend
CD40	PE	3/23	1:200	Biolegend
CD45	BV510	30F11	1:1000	Biolegend
CD80	APC	16-10A1	1:100	Biolegend
CD86	APC	GL-1	1:100	Biolegend
MHC class I	FITC	28-8-6	1:200	Biolegend
MHC class II	PE	AF6-120.1	1:1500	Biolegend
TLR4	PE-Cy7	SA15-21	1:500	Biolegend
PD-L1	PE	MIH5	1:100	BD Bioscience
Foxp3	PE-Cy5.5	FJK-16s	1:50	Invitrogen
CTLA-4	PE-Cy7	UC10-4B9	1:100	BioLegend

### OT-II antigen presentation assay

The midbrain and striatum from six mice were pooled, and cell suspensions were prepared with papain (Worthington) and DNase I (Sigma-Aldrich) as described above. The cells were incubated with antibodies against CD11b-PE (1:100, Miltenyi Biotec) and CD45-FITC (1:50, Miltenyi Biotec), and stained with 7-AAD (0.2 μg/mL, Invitrogen). Microglial cells (CD45^low^/CD11b^+^) were separated on a FACSAria IIu cell sorter (BD Biosciences). The spleen of one OT-II mice was processed with collagenase D and DNase I, and the isolated cells were incubated with anti-CD11c Microbeads (1:5, Miltenyi Biotec) to sort dendritic cells on an autoMACS Pro Separator (Miltenyi Biotec). The negative fraction was incubated with the CD4^+^ T cell Isolation Kit (1:5, Miltenyi Biotec), and CD4^+^ T cells were collected. These CD4^+^ cells were stained with 0.125 μM carboxyfluorescein succinimidyl ester (CFSE, Sigma) and co-cultured with antigen-presenting cells (APCs) at a ratio of 10:1 in the presence of the OVA_323–339_ peptide (10 μg/mL, Polypeptide, Strasbourg, France). After 7 days in culture, the cells were stained with Zombie NIR Viability Dye and the division of the CD4^+^ cells was analyzed on a FACSCanto II flow cytometer.

### Magnetic bead separation

Cellular suspensions from the striatum and midbrain were incubated with FcR Blocking Reagent (1:50, Miltenyi Biotec) and CD11b MicroBeads (1:10, Miltenyi Biotec). Microglial CD11b^+^ cells were separated on an autoMACS Pro Separator (Miltenyi Biotec); an aliquot of the separated cells was stained with CD11b-PE (1:100) and CD45-FITC (1:50, Miltenyi Biotec), and then analyzed on a BD FACSCanto II flow cytometer to determine their purity. Of the viable cells, 98% were microglia (CD45^low^CD11b^+^) and 2% infiltrated myeloid cells (CD45^high^CD11b^+^). After separation, the cells were pelleted, resuspended in the lysis/binding buffer from the Dynabeads mRNA Direct Kit (Ambion, Foster City, CA), and stored at − 80 °C for further processing.

### RNA sequencing

RNA-seq was performed using MARS-seq adapted for bulk RNA-seq [16, 17] with minor modifications. Briefly, poly-A RNA was extracted with Dynabeads Oligo (dT) (Thermo Fisher Scientific, Vilnius, Lithuania) and reverse-transcribed with AffinityScript Multiple Temperature Reverse Transcriptase (RT, Agilent) using poly-dT oligos (IDT) carrying a 7-bp index. Upon indexing, samples were pooled and subjected to linear amplification using HiScribe T7 High Yield RNA Synthesis Kit (New England Biolabs, Ipswich, MA, USA). The resulting antisense RNA was fragmented into 250–350 bp fragments using RNA Fragmentation Reagents (Thermo Fisher Scientific) and dephosphorylated for 15 min at 37 °C with 1 U FastAP (Thermo Fisher Scientific). Partial Illumina adaptor sequences [16] were ligated to the fragments with T4 RNA Ligase 1 (New England Biolabs), and reverse transcription was repeated. Full Illumina adaptor sequences were added during library amplification with KAPA HiFi DNA Polymerase (Kapa Biosystems, Wilmington, MA, USA). The libraries were then quantified using a Qubit 3.0 Fluorometer (Life Technologies, Carlsbad, CA, USA), and their size profiles were examined in an Agilent 4200 TapeStation System. Libraries were sequenced in an Illumina NextSeq 500 instrument at a sequence depth of 10 million reads per sample.

### Bioinformatics analysis

RNA sequencing data was analyzed using the following workflow: (i) verification of sample quality with the FastQC software, (ii) alignment of the reads to the mouse genome (GRCm38) using STAR [18], (iii) quantification of gene expression using read counts of exonic gene regions with featureCounts [19], (iv) gene annotation with Gencode M17 [20], and (v) statistical analysis of differential expression with R/Bioconductor [21]. The data are publicly available in the GEO database with the accession number GSE133617. Gene expression data was independently normalized with edgeR [22] and voom [23]. A filtering process was performed after quality assessment and outlier detection using R/Bioconductor [21]. The genes with less than six read counts in more than 50% of the samples of all the conditions studied (Str and Mdb) were considered not expressed. LIMMA (Linear Models for Microarray Data) [23] was used to identify the genes with significant differential expression between experimental conditions. Genes were selected as differentially expressed using a cutoff *p* value of < 0.01. Functional and clustering analyses, and graphical representations, were obtained using R/Bioconductor [21].

### RNA extraction from tissue and semi-quantitative real-time PCR

Brain regions of interest were dissected on ice, fast frozen in liquid nitrogen, and stored at − 80 °C. Total RNA was extracted using the TRI Reagent (Sigma-Aldrich) according to the manufacturer’s instructions. After treatment with 1 U of DNase I (Thermo Fisher Scientific), reverse transcription of 2 μg of total RNA was performed using 200 U of SuperScript IV (Invitrogen) and 100 ng of random hexamer oligodeoxyribonucleotides (Invitrogen) in a final volume of 20 μL. The mRNA expression was studied by semi-quantitative real-time PCR using iQ SYBR Green Supermix (Bio-Rad, Hercules, CA, USA) in a CFX96 Touch real-time detection system (Bio-Rad). The following primers were used: TNFα forward-TGCCTATGTCTCAGCCTCTT, TNFα reverse-TGATGAGAGGGAGGCCATTT; IL1β forward-TGAAATGCCACCTTTTGACA, IL1β reverse-AGCTTCTCCACAGCCACAAT; IL6 forward-GTTCTCTGGGAAATCGTGGA, IL6 reverse-TCCAGTTTGGTAGCATCCATC; IL10 forward-CCAAGCCTTATCGGAAATGA, IL10 reverse-TTTTCACAGGGGAGAAATCG; TGFβ forward-CGGCAGCTGTACATTGAC, TGFβ reverse-TCAGCTGCACTTGCAGGAGC; and GAPDH forward-GTTCCAGTATGACTCCACTCAC, GAPDH reverse-GGCCTCACCCCATTTG. Each PCR was carried out in duplicates to obtain an average Ct value. The results were normalized to GAPDH, and the amount of each transcript was expressed as 2^ΔCt^ (ΔCt = Ct [GAPDH] − Ct [gene]). The relative expression of each gene was normalized with the mean of control animals to detect changes with respect to untreated animals.

### Immunofluorescence

Mice were anesthetized with ketamine/xylazine and perfused transcardially with Ringer’s solution and 4% paraformaldehyde in PBS. Immunofluorescence was performed in 40-μm-thick free-floating sections, blocking the tissue in 4% normal goat serum, 0.05% Triton TX-100 (Sigma-Aldrich), and 4% bovine serum albumin (Sigma-Aldrich) in PBS. Sections were incubated overnight at room temperature with the primary anti-Iba1 rabbit antiserum (1:1000; Wako, Osaka, Japan), and antibody binding was detected using an Alexa Fluor 633-conjugated goat anti-rabbit antibody (1:1000; Invitrogen). Sections were finally stained with DAPI (1:50,000; Sigma-Aldrich), mounted on glass slides in a 0.2% solution of gelatin in 0.05 M Tris-HCl buffer, and dried and then dehydrated in xylene (Panreac, Barcelona, Spain) for 12 min before coverslipping in DPX (VWR, Leuven, Belgium). Tissue sections were visualized in a confocal laser scanning microscope LSM800 with Airyscan (Zeiss, Oberkochen, Germany). Images were acquired using a × 63 oil objective with constant microscope parameters and laser intensity. A projection stack of 10 images per slice is shown.

### Statistical analysis

Statistical analyses were performed with Stata 12.1. Flow cytometry data were analyzed by two-way ANOVA; significant *p* interaction values (*P*_int_) are given in the text. Since not all the data followed a normal distribution, the results obtained were validated with a permutation test (Per test). The effect of LPS in each region was analyzed with a contrast analysis with the Šidák correction. One-way ANOVA followed by multiple pairwise comparisons with the Šidák correction was used to determine the differences between regions. In all instances, *p* values < 0.05 were considered as statistically significant. The graphs were prepared with GraphPad Prism 5 (La Jolla, CA, USA) and represent the means ± 95% CI. Data obtained from real-time PCR reactions were analyzed using the median test. In these graphs, the median with the interquartile range is represented.

## Results

### Morphological changes of microglia in response to systemic LPS stimulation

**Fig. 1:**
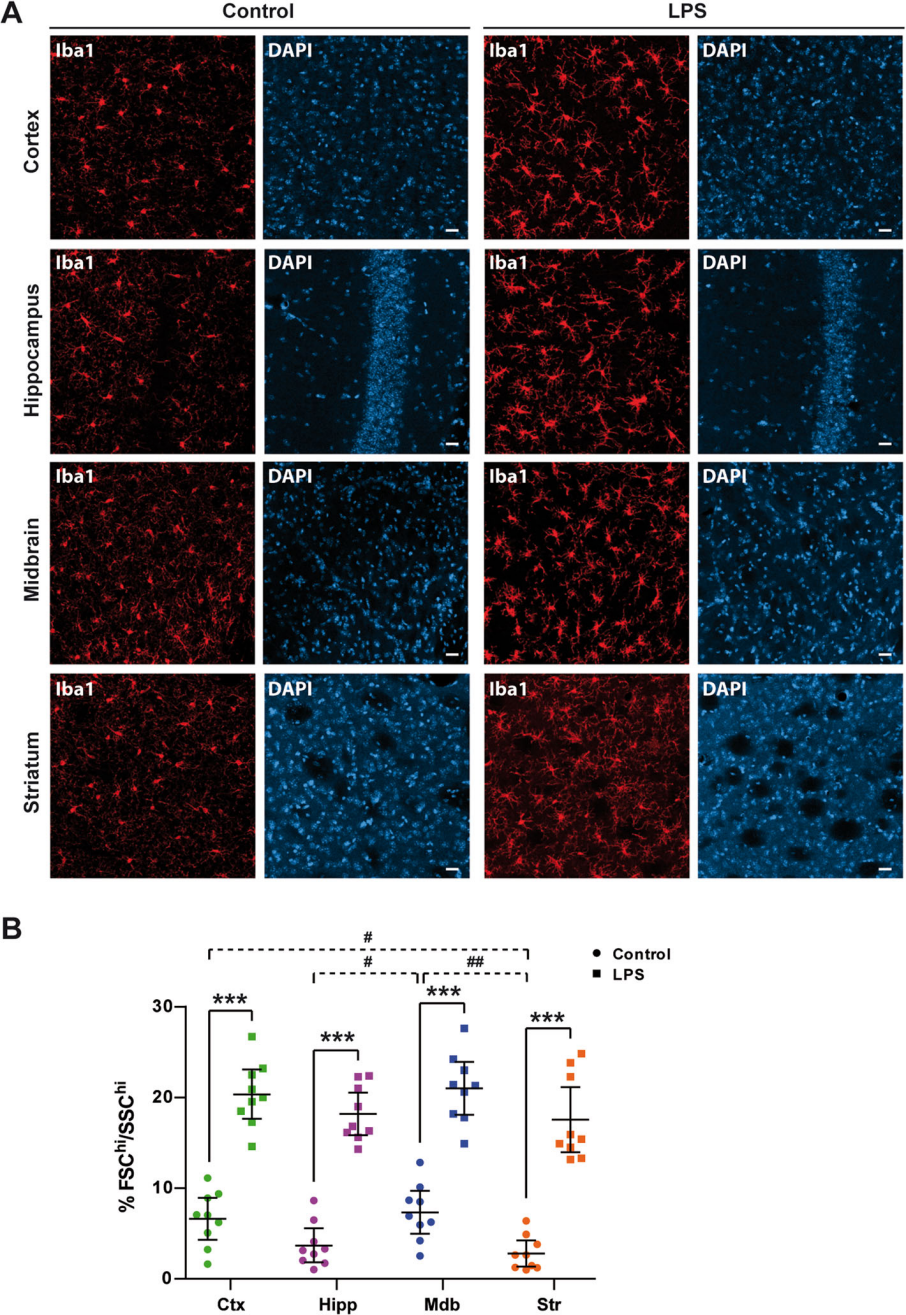
LPS affects the size and complexity of microglial cells. **a** Representative images showing Iba-1 immunoreactivity in the cortex (Ctx), hippocampus (Hipp), midbrain (Mdb), and striatum (Str) of control and LPS-treated mice. **b** The size and complexity of the cells were measured by flow cytometry based on the forward scatter (FSC) and side scatter (SSC), respectively. Microglial cells were purified from the Ctx, Hipp, Mdb, and Str of saline and LPS-treated mice, and the data from nine animals per group are represented with their mean ± 95% CI. Two-way ANOVA followed by contrast test with Šidák adjustment was used to determine LPS effects in each region (***p < 0.001). One-way ANOVA was used to analyze the region effects in control animals (^#^*p* < 0.05, ^##^*p* < 0.01). Scale bar 20 μm

To study whether the regional heterogeneity of microglia could affect how these cells modulate the immune response, we induced a systemic inflammatory reaction in mice by means of intraperitoneal injection of LPS. LPS can target brain microglia either directly or through the production of pro-inflammatory cytokines that cross the blood-brain barrier (BBB) [24–28]. Microglia can transit from a surveying phenotype to an activated state, accompanied by morphological changes [11]. Immunostaining of the cortex, hippocampus, midbrain, and striatum with Iba1 showed how LPS provoked a marked modification in the shape of microglia (Fig. 1a). Adult mouse microglial cells were purified on a density gradient, and the single cell suspension obtained was analyzed by fluorescence-activated cell sorting (FACS), identifying microglia as the CD45^low^CD11b^+^ cell population (see Additional file 1: Figure S2 for a representative gating strategy). Analysis of the forward (FSC) and side scatter (SSC) parameters gives an idea of the size and internal complexity of the cells, respectively. Under steady-state conditions, midbrain microglia presented significantly higher FSC and SSC values than cells from the hippocampus or striatum, and similar to those of the cortex (Fig. 1b). Systemic administration of LPS induced a significant increase in cell size and complexity in microglia (Fig. 1b). The induction of morphological changes in microglia indicates that peripheral administration of LPS induces a transition between different states of microglial activation. The higher FCS and SSC values of cortical and midbrain microglia might reflect a different activation profile under basal conditions, highlighting the regional heterogeneity of microglial cells.

### LPS induces a specific expression of anti-inflammatory cytokines in the midbrain

**Fig. 2:**
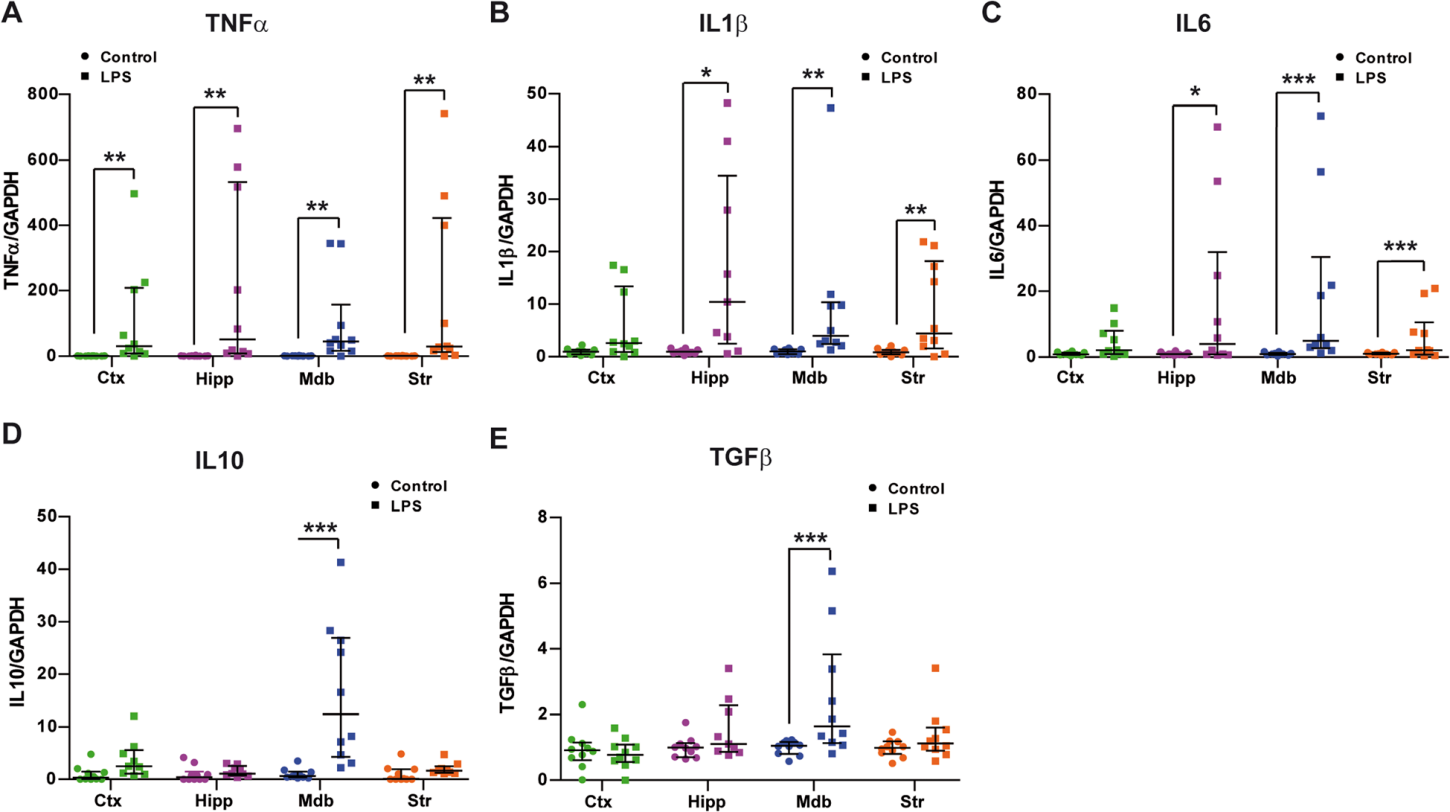
Effect of LPS on cytokine expression. Real-time PCR for TNFα (**a**), IL1β (**b**), IL6 (**c**), IL10 (**d**), and TGFβ (**e**) from the cortex (Ctx), hippocampus (Hipp), midbrain (Mdb), and striatum (Str) of control and LPS-treated animals. The results are expressed as the ratio to the control group. Median with interquartile range from ten animals per group is represented. The effect of LPS was analyzed pair by pair with the median test: *p < 0.05, **p < 0.01, ***p < 0.001

As part of their activation process, microglia secrete a wide range of soluble factors that can modulate the local immune response [29]. We assessed the cytokine expression induced by LPS in specific regions by real-time PCR. Analyses of the relative expression of pro-inflammatory cytokines after LPS administration showed a significant increase in TNFα (Fig. 2a), IL1β (Fig. 2b), and IL6 (Fig. 2c) mRNA in the hippocampus, midbrain, and striatum, while in the cortex, only TNFα expression was significantly higher. By contrast, the expression of the anti-inflammatory cytokines IL10 (Fig. 2d) and TGFβ (Fig. 2e) were significantly elevated exclusively in the midbrain. Hence, the systemic administration of LPS appears to alter the local production of cytokines in the brain, inducing a broad elevation of pro-inflammatory cytokine levels that affects at least the four areas studied here and an increase in the anti-inflammatory cytokines which is apparently exclusive to the midbrain (Table 2).

**Table 2:** Summary of the results obtained in the different brain regions after LPS administration

	Ctx	Hipp	Mdb	Str
TNFα	↑	↑	↑	↑
IL1β	=	↑	↑	↑
IL6	=	↑	↑	↑
IL10	=	=	↑	=
TGFβ	=	=	↑	=
TLR4	↑	↑	=	↑
CD40	↑	↑	↑	↑
MHC-I	↑	↑	↑	↑
MHC-II	=	=	↓	=
CD86	↑	↑	=	↑
CD80	↑	↑	↑	↑

“↑,” significant increase; “↓,” significant decrease; “=,” no significant changes with respect to the control group of animals

### Microglial inflammatory response to LPS is different in the midbrain

**Fig. 3:**
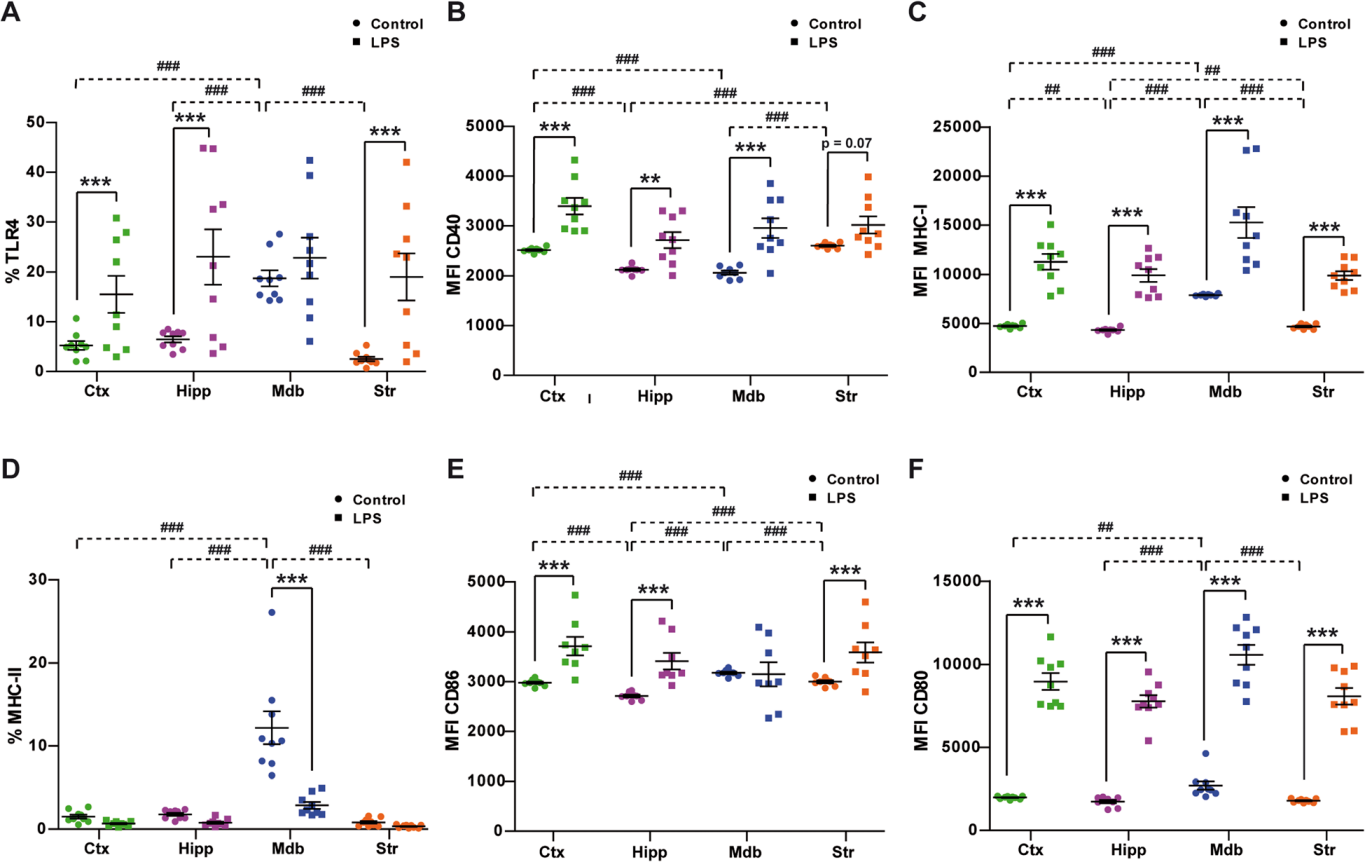
Analysis of inflammatory cell surface markers on microglial cells after LPS exposure. Microglial cells were purified from the cortex (Ctx), hippocampus (Hipp), midbrain (Mdb), and striatum (Str) from control and LPS-treated mice. The expression of inflammatory cell surface markers was assayed in the CD45^low^CD11b^+^ fraction of cells by flow cytometry. Data obtained were analyzed by two-way ANOVA followed by pairwise comparisons with Šidák adjustment to determine the effect of LPS in each region. Significant *p* values for interaction (*P*_int_) were validated with a permutation test (Per test). The region effect in control animals was analyzed by one-way ANOVA followed by a contrast analysis with Šidák adjustment. **a** Percentage of CD45^low^CD11b^+^ cells expressing TLR4, *P*_int_ < 0.001, Per test 95% CI 0–0.004. **b** MFI of CD40. **c** MFI of MHC-I. **d** Percentage of MHC-II cells, *P*_int_ < 0.001, Per test 95% CI 0–0.004. **e** MFI of CD86, *P*_int_ < 0.001, Per test 95% CI 0–0.003. **f** MFI of CD86. The data are from eight to nine animals per group and are represented as the mean ± 95% CI. Significant effect of LPS in each region: **p < 0.01, ***p < 0.001. Significant differences between regions in control animals: ^##^*p* < 0.01, ^###^*p* < 0.001

Following any perturbation, adult resting microglia are rapidly activated and they express a wide range of cell surface molecules related to the immune response. The expression of some of these membrane proteins was analyzed by FACS in CD45^low^CD11b^+^ cell suspensions. TLR4 is activated by LPS and neuronal damage, and it modulates autophagy in microglial cells [3, 5, 30, 31]. LPS administration affected TLR4 expression distinctly in different regions (Fig. 3a, *F*_3,48_ = 6.55, *P*_int_ < 0.001). Under basal conditions, a small fraction of microglial cells from the cortex (5.2% ± 0.9), hippocampus (6.5% ± 0.6), and striatum (2.5% ± 0.4) expressed TLR4, whereas this subpopulation represented a significantly higher proportion of the microglia in the midbrain (18.8% ± 4.8, Fig. 3a). Peripheral LPS administration provoked a significant increase in the fraction of cells that expressed TLR4 in the cortex, hippocampus, and striatum, while this population remained constant in the midbrain (Fig. 3a). Hence, TLR4 expression appeared to be selectively upregulated by LPS in those regions with a lower basal TLR4 expression. In a healthy nervous system, the constitutive expression of CD40 by microglia is relatively low, yet under inflammatory conditions, CD40 expression is enhanced and the interaction with its ligand CD40L is one of the multiple signals necessary for a productive immune response [32, 33]. In our system, LPS induced a significant increase in the cell surface expression of CD40 by microglia in the different regions analyzed (Fig. 3b). However, the basal expression of CD40 on microglial cells was significantly higher in the cortex and striatum than in the hippocampus and midbrain (Fig. 3b). These results reflect a general pro-inflammatory effect of LPS across the different regions and emphasize the local heterogeneity of microglial cells.

Microglia are the main resident APCs in the brain parenchyma, and thus, we explored whether this property was altered locally by LPS. Under basal conditions, midbrain microglia presented a significantly higher density of MHC-I molecules than the rest of the regions analyzed (Fig. 3c). However, LPS administration induced a significant increase in the median fluorescence intensity (MFI) of MHC-I expression in microglial cells that was similar in all regions (Fig. 3c). MHC-II was differentially expressed by microglial cells under physiological conditions and after LPS administration (Fig. 3d; *F*_3,48_ = 16.9, *P*_int_ < 0.001). In steady-state conditions, microglial cells expressing MHC-II were almost undetectable in the cortex (1.5 ± 0.2%), hippocampus (1.8 ± 0.2%), and striatum (0.8 ± 0.2), yet this cell subpopulation was significantly more abundant in the midbrain (12.2 ± 5.9%). Moreover, no overlap was observed between the TLR4^+^ and MHC-II^+^ microglial subpopulations in the midbrain (Additional file 1: Figure S3). LPS induced a robust reduction in the cell surface expression of MHC-II by microglial cells in the midbrain, and this microglial subpopulation decreased significantly to 2.9 ± 0.4 (Fig. 3d). The basal expression of CD86 varied significantly in the different brain regions studied, and LPS produced a distinct effect on CD86 MFI (Fig. 3e; *F*_3,42_ = 13.7, *P*_int_ < 0.001). The midbrain presented the highest intensity of CD86, and the hippocampus the lowest. LPS produced a significant increase in the CD86 MFI in the cortex, hippocampus, and striatum but not in the midbrain (Fig. 3e). The microglia in the midbrain of control animals presented a significantly higher density of CD80 than in the other regions analyzed (Fig. 3f). Systemic administration of LPS induced a significant increase in the CD80 intensity on the cell surface of microglial cells, which was similar in all the regions analyzed (Fig. 3f). These results, summarized in Table 2, show that LPS induced the expression of the cell-surface pro-inflammatory molecules CD40, MHC-I, and CD80 in all regions studied. A specific effect characterized by the lack of changes in TLR4 and CD86 and by the decrease in MHC-II expression was observed in the midbrain.

**Fig. 4:**
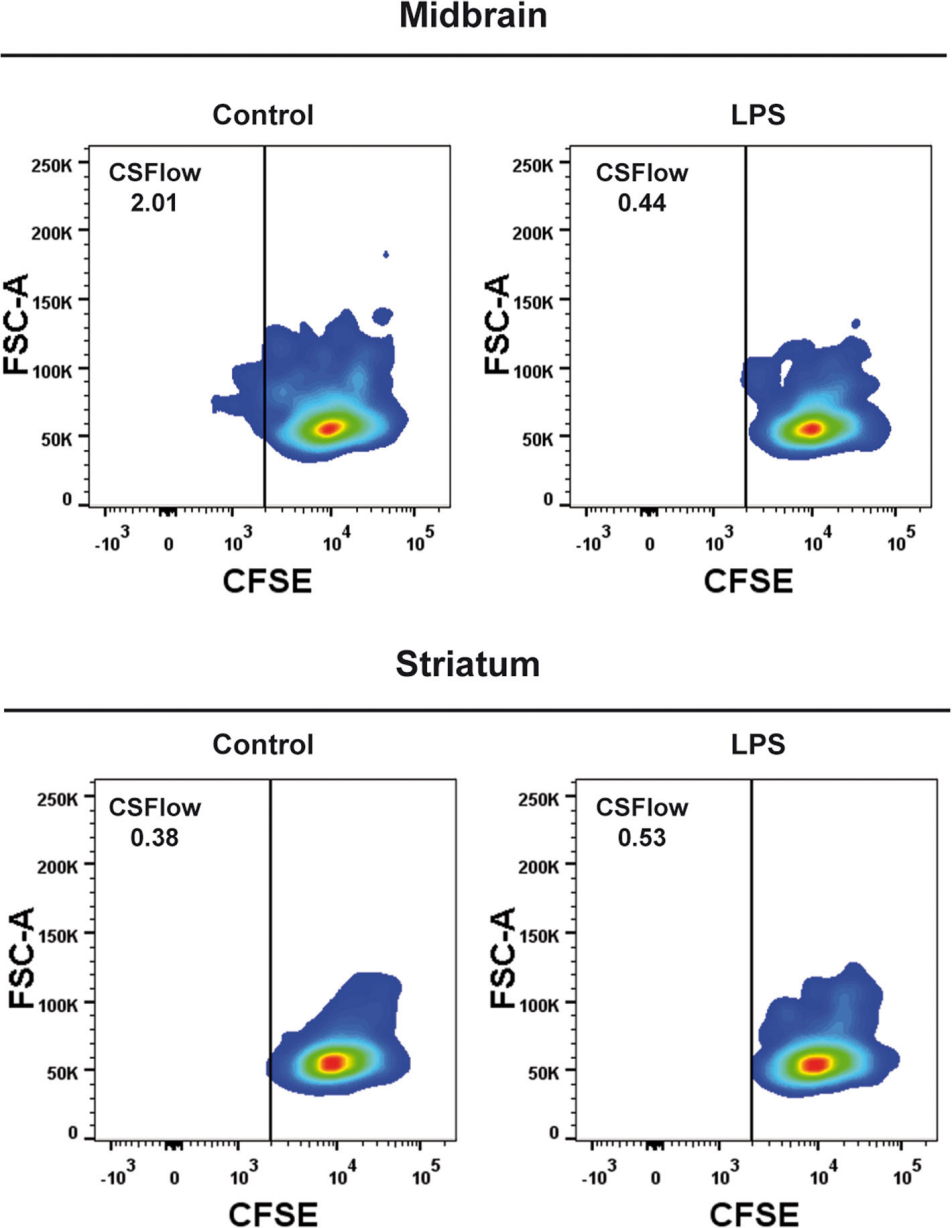
Antigen-presenting capacity of microglia from the midbrain and striatum. Representative density plots from one experiment of flow cytometry analysis of CFSE-stained CD4^+^ T cells co-cultured during 7 days with microglia are shown. Microglial cells were purified from the midbrain (upper row) or the striatum (lower row) of control and LPS-treated mice. The fraction of CD4^+^ T cells that proliferated and showed a low CFSE staining is included in each plot. Carboxyfluorescein succinimidyl ester; FSC, forward scatter

We further explored the functional antigen-presenting capacity of these cells by sorting microglial cells from the midbrain and the striatum of control and LPS-treated animals, and co-culturing them with CFSE-labeled CD4^+^ OT-II T cells in the presence of the class II (I-A^b^)-restricted epitope of ovalbumin (Fig. 4). Spleen dendritic cells (CD11c^+^) were used as positive controls in these studies (Additional file 1: Figure S4). Microglia from the midbrain of naïve animals induced weak, yet detectable, antigen-specific proliferation of CD4^+^ T cells (1.2 ± 0.77% CSFE^low^). By contrast, LPS-primed midbrain microglia failed to induce in vitro T cell proliferation (0.5 ± 0.04% CSFE^low^; Fig. 4), as did striatal microglia from either control (0.4 ± 0.08% CSFE^low^) or LPS-treated mice (0.5 ± 0.31% CSFE^low^; Fig. 4). These results are consistent with the expression of MHC-II by microglial cells from these two brain regions in control and LPS-treated mice. Our data demonstrate that a unique microglial cell subpopulation with antigen-presenting properties exists in the midbrain but not in other brain regions. Interestingly, under inflammatory conditions, the MHC-II expression of this subset of cells is downregulated and may control the T cell response.

### Differences in the midbrain and striatum microglial transcriptome

The expression of cell surface markers of inflammatory responses under basal conditions was significantly stronger in the midbrain compared to the striatum, suggesting that midbrain microglia adopt a more vigilant immune alert status than striatal populations. To further explore this possibility, the microglial transcriptome from these regions was analyzed in single cell density gradient and immuno-magnetic purified (based on CD11b antigen expression) microglial suspensions from the striatum and midbrain. Cell purity and viability were assessed by FACS, and 97% of cells were CD45^low^CD11b^+^ cells in the positive fraction and a few CD45^high^CD11b^+^ cells (3%).

**Fig. 5:**
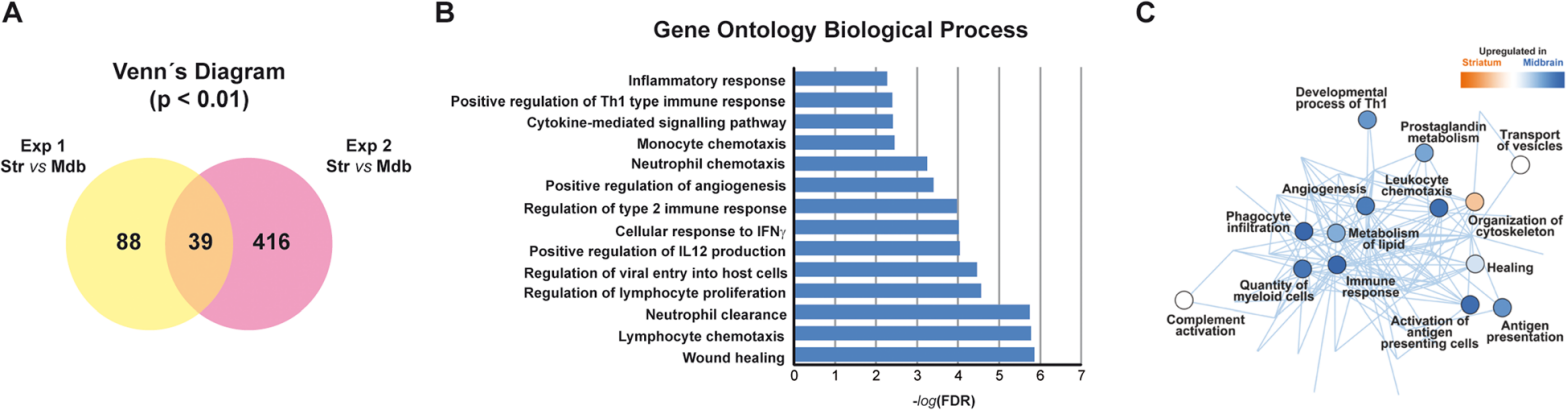
Transcriptome analysis of microglial cells purified from the striatum and midbrain. The data were obtained from two independent experiments, the first (Exp 1) with *N* = 2 mice and the second one (Exp 2) with *N* = 3 mice. **a** Venn diagram of the genes differentially expressed between the striatum (Str) and midbrain (Mdb) in the two independent experiments (*p* < 0.01). The intersection shows the common genes (39) expressed differentially in the 2 experiments. **b** Enrichment analysis of Gene Ontology biological processes of genes overexpressed in the midbrain using PANTHER. Overrepresentation test and Fisher’s Exact test with a false discovery rate (FDR < 0.01) multiple test correction. **c** Ingenuity Pathway Analysis of functions regulated by the differentially expressed genes. The intensity of the circle’s color reflects activation or inhibition intensity based on the *z*-scores, and the nodes represent the genes and their connections to functions

The midbrain and striatal microglial transcriptome of 5 mice was examined in two independent experiments using a protocol of single cell RNAseq. The gene expression profile of adult microglia in the healthy striatum and midbrain was heterogeneous since a marked inter-experimental variability was observed, probably due to the immune nature of microglial cells. The overlap in the genes differentially expressed between the striatum and midbrain in the two experiments was 39 (Fig. 5a, Additional file 1: Table S1), all of which were overexpressed in the midbrain suggesting that they might have additional functions to those of striatal cells (Fig. 5a), such as the H2-Ab1 gene that belongs to the MHC-II complex (Additional file 1: Table S1). An analysis of biological processes through Gene Ontology revealed that the immune response was significantly overrepresented in these transcriptomes, with multiple processes associated with several aspects of immune function (Fig. 5b). The network generated with Ingenuity Pathway Analysis software also showed an enhanced immune response in the microglia from the midbrain, with different nodes related to the interaction between microglia and other elements of the immune system (Fig. 5c). Thus, the regional diversity between these two structures would mainly focus on their interaction with the immune system and their ability to modulate the immune response, confirming our previous observations.

### Heterogeneous lymphocyte populations in the brain under inflammatory conditions

**Fig. 6:**
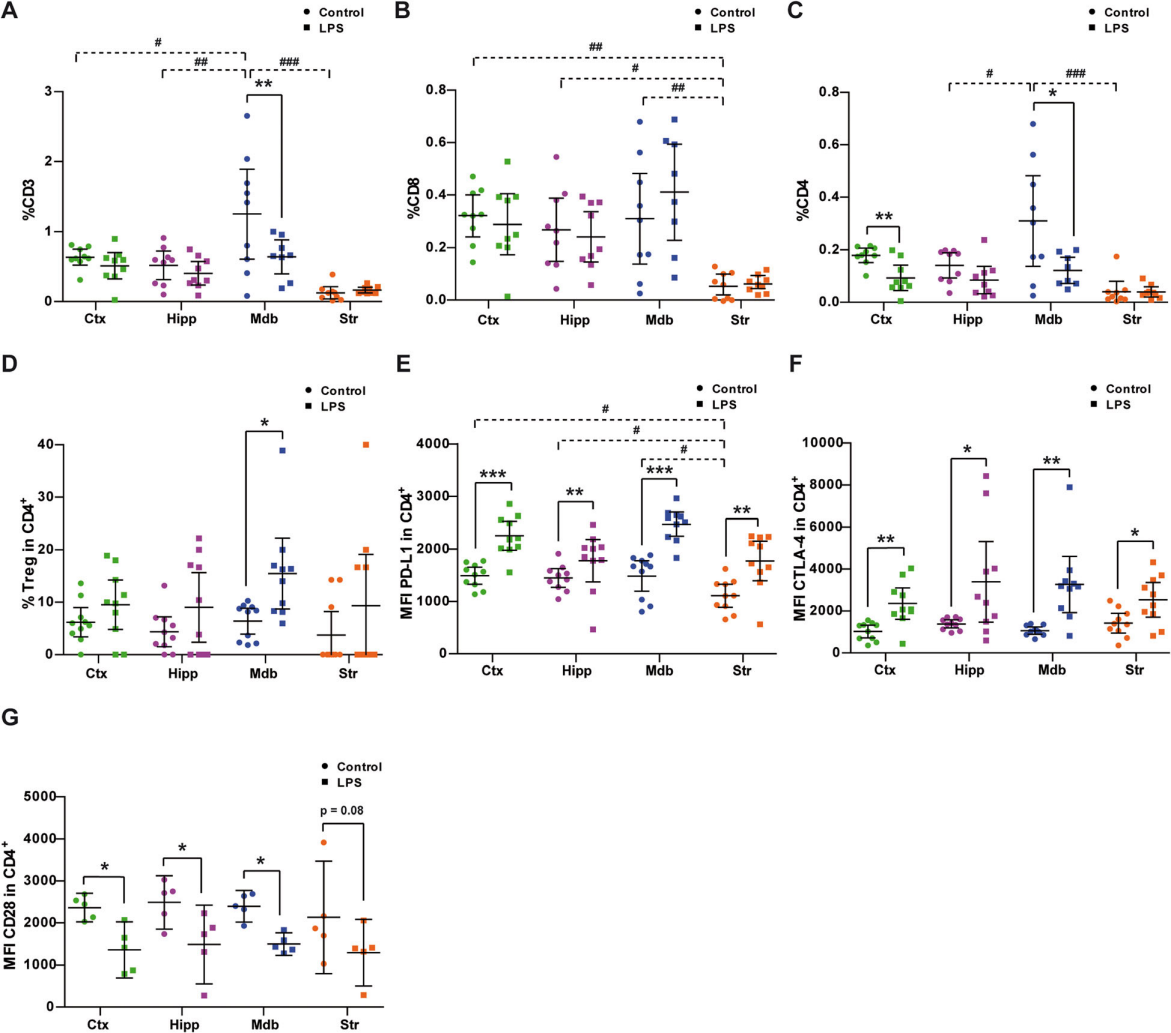
Rearrangement of the T cell subpopulations upon LPS stimulation. Cells were purified from the cortex (Ctx), hippocampus (Hipp), midbrain (Mdb), and striatum (Str). The expression of lymphocyte cell surface markers was analyzed by flow cytometry, and data were analyzed by two-way ANOVA followed by pairwise comparisons with Šidák adjustment to determine the effect of LPS in each region. Significant *p* values for interaction (*P*_int_) were validated with a permutation test (Per test). The region effect in control animals was analyzed by one-way ANOVA followed by a contrast analysis with Šidák adjustment. **a** CD3^+^, *P*_int_ = 0.03, Per test 95% CI 0.022–0.045; **b** CD8^+^; **c** CD4^+^, *P*_int_ = 0.03, Per test 95% CI 0.019–0.041; **d** CD4^+^CD25^+^Foxp3^+^ (Treg) lymphocytes; **e** PD-L1; **f** CTLA-4; and **g** CD28 performed on single cell suspensions from control and LPS-treated mice. The results are represented as the percentage of positive cells from the total viable cells isolated (**a**–**c**), as the percentage of the total CD4^+^ cells (**d**), and as the MFI in CD4^+^ cells (**e**–**g**). The data from eight to nine (**a**–**f**) and five (**g**) animals per group are shown as the mean ± 95% CI. Significant effect of LPS in each region: *p < 0.05, **p < 0.01. Significant differences between regions in control animals: ^#^*p* < 0.05, ^##^*p* < 0.01, ^###^*p* < 0.01

To determine whether the inflammatory status affects lymphocyte infiltration in the different regions analyzed, we assessed lymphocyte subpopulations within the cell suspensions obtained from the different brain regions. The fraction of CD3^+^ cells (Fig. 6a) was significantly higher in the midbrain (1.3% ± 0.3) than in the cortex (0.6% ± 0.05), hippocampus (0.5% ± 0.08), or striatum (0.1% ± 0.04), and LPS administration significantly diminished the CD3^+^ cells in the midbrain but not in the other regions studied (Fig. 6a; F_3,47_ = 3.2, *p* = 0.03). The relative amount of CD8^+^ was not affected by LPS administration, and it was similar in all areas, except in the striatum where there were significantly fewer cells (Fig. 6b). The midbrain was enriched in CD4^+^ cells relative to the hippocampus and striatum (Fig. 6c), indicating that the increase in CD3^+^ expression would be a result of enhancing this population of cells. Administration of LPS significantly reduced the CD4^+^ cells in the midbrain and cortex (Fig. 6c; *F*_3,47_ = 3.3, *p* = 0.03). These results indicate that modulation of the inflammatory signals in the brain by LPS mainly affects CD4^+^ lymphocytes, which may play a relevant role in the control of the inflammatory response and would be consistent with the decrease in MHC-II expression in the midbrain microglia. The elevated levels of IL10 and TGFβ transcripts in the midbrain of LPS-treated animals led us to explore the presence of CD4^+^CD25^+^Foxp3^+^ (Treg) lymphocytes. This subset of Treg cells was also specifically enhanced in the midbrain (Fig. 6d; *F*_3,54_ = 8.7, *p* = 0.009), suggesting a robust immunosuppressor response exclusive to this region.

CD80 is mainly expressed by APCs, and it is the receptor for CD28 and CTLA-4 found on the surface of T cells. CD80 also binds to the PD-L1 present in APCs and in activated T cells. In concert with MHC molecules, CD80 transmits an activating signal to T cells through its interaction with CD28. However, CD80 also regulates the immune system through an inhibitory interaction with CTLA-4 and PD-L1 on T cells [34, 35]. The opposite expression of MHC-II (downregulated) and CD80 (upregulated) in the midbrain microglia from LPS-treated mice suggests an inhibitory role for CD80 molecule in this cell subset under inflammatory conditions. Examination of CD4^+^ lymphocyte subpopulations revealed that LPS administration significantly increased the expression of PD-L1 in this T cell subset in all brain areas (Fig. 6e; *F*_3,53_ = 69.1, *p* < 0.001), whereas naïve animals had significantly weaker expression of PD-L1 in the striatum than in the other brain regions tested (Fig. 6e; *F*_3,53_ = 9.1, *p* < 0.001). Similarly, a general increase in the levels of CTLA-4 expression by CD4^+^ lymphocytes was detected after LPS treatment (Fig. 6f; *F*_3,54_ = 16.2, *p* < 0.001). By contrast, CD28 expression was significantly reduced in CD4^+^ T cells present in these regions (Fig. 6g; *F*_3,24_ = 15.2, *p* = 0.004). Together, these results show that the immune response in the brain upon LPS challenge, and mainly that in the midbrain, seems to be dampened, as suggested by the decrease in the number of infiltrating effector T cells, especially that of CD4^+^ T cells. This is also consistent with the enhanced expression of co-inhibitory receptors on the remaining T cells like CTLA-4 and PD-L1, the increase in the proportion of Tregs, and the enhanced production of anti-inflammatory cytokines.

## Discussion

**Fig. 7:**
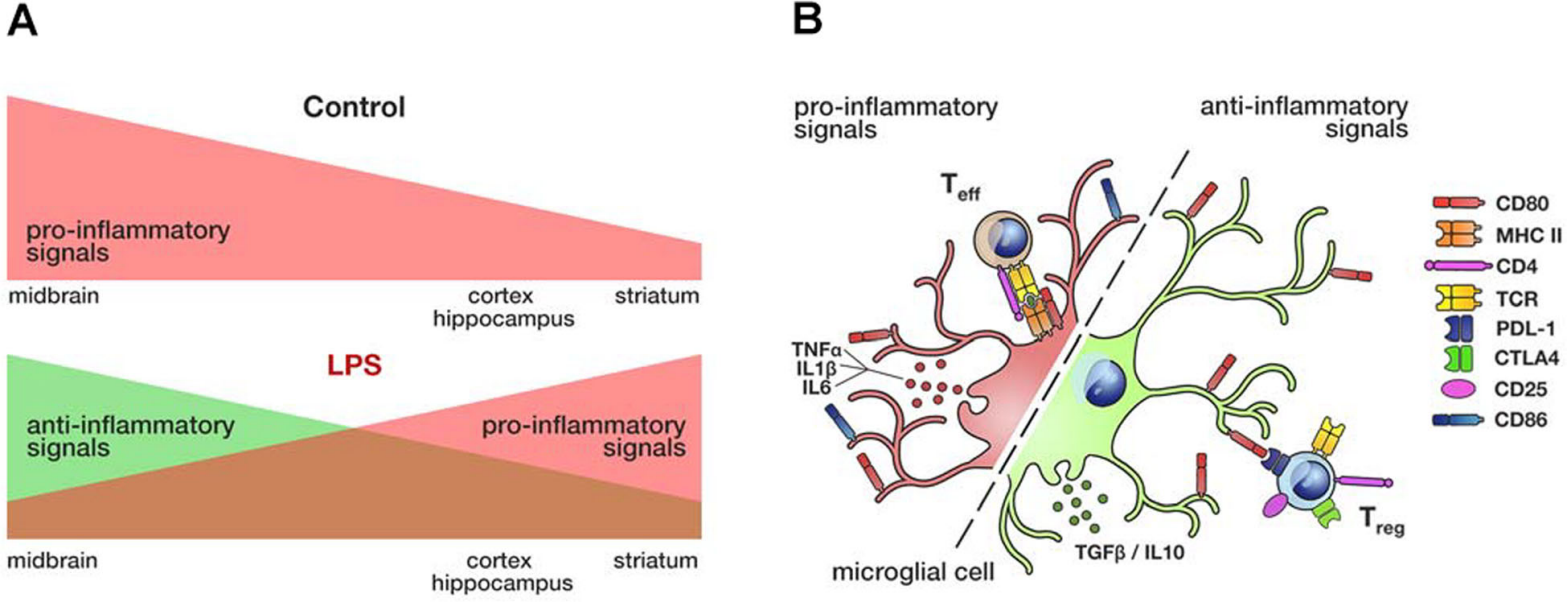
Unique properties and specific responses of midbrain microglia to LPS. **a** Microglial cells from the midbrain are balanced towards a primed state, a constitutive state of immune alert compared to microglia from other brain regions. Upon LPS stimulation, microglia from the midbrain switch to a potent immunosuppressive phenotype. This response differs from that observed in the striatum, cortex, or hippocampus. **b** Pro-inflammatory phenotype of microglial cells includes stronger MHC-II, CD80, and CD86 expression, and better antigen-presenting capability and T cell recruitment. These changes are mediated or accompanied by stronger production of the classical pro-inflammatory cytokines. The anti-inflammatory phenotype of microglial cells is characterized by the downregulation of MHC-II expression and consequently the loss of antigen-presenting capability. Immunosuppressive microglia express high levels of CD80 without any change in their CD86 expression. CD80 may counteract the CD4 T cell response through its interaction with CTLA-4 and PD-L1 on activated T cells. The recruitment of Tregs and the production of immunosuppressive cytokines are also indicators of this anti-inflammatory state

There is growing evidence that adult microglia constitute a heterogeneous population of cells showing regional differences [14, 36], potentially responding distinctly to either injury or damage. In this study, we show that under basal conditions, the midbrain microglia present an immune alert state and unique subpopulations of cells which were not evident in other brain regions. Systemic administration of LPS induced region-specific changes that were related to the basal inflammatory tone. In the midbrain, the region with the strongest immune surveillance, LPS induced a unique anti-inflammatory effect that was not observed in the cortex, hippocampus, or striatum. Our results suggest that the inflammatory tone depends on the context determining the response to an inflammatory signal like LPS (Fig. 7).

Characterizing the immune phenotype of microglia from different brain regions revealed that steady-state microglia in the midbrain present a higher immune alertness than those in the striatum, cortex, or hippocampus. Two unique fractions of microglial cells were identified in the midbrain, one expressing TLR4 and the other MHC-II. TLRs recognize damage-associated molecular patterns produced by dead cells eliciting a sterile inflammatory response [3–5]. The strong expression of MHC class I and II molecules, as well as accessory molecules for antigen presentation like CD80 and CD86 [11], indicates that the midbrain contains cells capable of carrying out specific functions related to the immune response. Specifically, we demonstrated that the MHC-II^+^ midbrain microglia are functional APCs within the CNS, an activity that was absent in the striatum, and given the extent of MHC-II expression, probably in the cortex and hippocampus as well. However, microglial cells are poor APCs relative to spleen dendritic cells. Accordingly, a higher concentration of CD4^+^ T lymphocytes was observed in the midbrain suggesting that an interplay between microglia and CD4^+^ T cells might occur in this region. Microglia with antigen-presenting properties are a rare cell type in the brain parenchyma, although a subpopulation of these cells was identified in the olfactory bulb after LPS stimulation [37]. Regionally heterogeneous gene expression profiles show that the transcriptome of cortical and striatal microglia differs from that of hippocampal microglia in genes involved in the immune response and in antigen presentation [14]. The data obtained from the midbrain was predominantly derived from SN pars reticulata (SNr) microglia, as well as SN pars compacta and VTA microglia. Microglial cells from the SNr may be assumed to be the main contributors to this pro-inflammatory state. In fact, they exhibited unique membrane properties when compared to VTA and SNc microglia [15], suggesting an even greater variability that might be driven by the GABAergic or dopaminergic environment. Together, these results highlight the microglial heterogeneity in the brain and point towards different functions under pathological conditions.

Modification of the inflammatory environment by peripheral administration of LPS triggers a general pro-inflammatory response that reaches the CNS [24–28]. Using different approaches, we demonstrate that LPS elicits a heterogeneous response in different brain regions. A context-dependent response of microglia to LPS has been described in the cortex and olfactory bulbs, even in the absence of morphological changes [37]. Flow cytometry analysis of thousands of CD45^low^CD11b^+^ cells indicated that the fraction of FCS^hi^/SSC^hi^ microglia increases in all brain regions (from ≈ 5 to ≈ 20%), suggesting that approximately 15% of the cells in the brain parenchyma experienced morphological changes and do not respond homogenously to LPS. The ensuing flow cytometry analysis could not associate any specific phenotype to the FCS^hi^/SSC^hi^ cells, concluding that the changes in cell size and complexity induced by LPS do not necessarily reflect an immune response of microglial cells. Several hypotheses could explain this observation: (i) by flow cytometry, we have analyzed specific aspects of the immune response, and these changes in FCS^hi^/SSC^hi^ might reflect specific immune functions not analyzed in this study, i.e., phagocytic microglia; (ii) they could be proliferating cells; (iii) they could reflect movement, static vs migrating cells; or (iv) they might be infiltrated macrophages.

LPS induced the production of pro-inflammatory cytokines (TNFα, ILβ, and IL6) and the elevation of inflammatory cell surface markers (CD40, MHC-I, and CD80) in the brain regions studied. Usually, MHC-II and CD80 expression goes hand in hand with the response to inflammatory stimuli, but we failed to detect an increase in MHC-II. CD80 can transmit an activating signal to T cells through its interaction with CD28, yet it also can mediate an inhibitory response by triggering CTLA-4 and PD-L1 on activated T cells [34, 35]. The enhanced PD-L1 and CTLA-4 expression and the decreased CD28 expression in CD4^+^ lymphocytes suggested that the microglial expression of CD80 was a general mechanism to counteract CD4^+^ T cell activation, also prompted by the lack of concomitant changes in MHC-II. Interestingly, LPS induced a specific anti-inflammatory effect in the midbrain. The increase of IL10 and TGFβ transcripts, two immunosuppressive cytokines that can counteract LPS or neurodegenerative damage in different experimental models [38–42], could reflect a protective reaction of the midbrain to an exacerbated inflammatory response aimed to prevent cell damage. In addition, they are key factors for the Treg lymphocyte responses and for the inhibition of effector T cells and APCs [43, 44]. The unaltered expression of the pro-inflammatory molecules TLR4 and CD86, the decrease in MHC-II^+^ microglial cells with the consequent loss of their antigen-presenting properties, and the enhanced presence of Tregs would contribute to repress the LPS-mediated immune response in the midbrain. These unique features instill a specific immune state that affects microglia and infiltrated CD4^+^ T cells, generating a specific response to LPS that resembles some of the strategies that cancer cells use to escape immune surveillance [35, 44].

In PD, chronic inflammation concurs with dopaminergic neurodegeneration in the SNc and it is likely to influence disease progression [45, 46]. The genetic association of PD with the human leukocyte antigen that encodes part of the MHC-II complex, and with the TNFα, IL6, and IL1 receptor genes, supports the involvement of the immune system and offers new targets to develop novel therapies [47–50]. In vivo imaging studies of microglial activation in PD show an early, widespread activation that remains stable over the course of the disease, without correlating with [^18^F]-dopa uptake in the putamen (equivalent to the caudate/putamen in rodents) [51]. In humans, dopaminergic terminal degeneration in the putamen precedes the loss of cell bodies in the SN [52]. Therefore, the regional activation of microglia early in the disease might differ in the putamen and SN, compromising dopaminergic neuron survival. Dopamine neurons are particularly sensitive to inflammation and present a high susceptibility to infiltrated T cells compared to neighboring and more resistant GABAergic neurons [53]. The high immune alert state of the midbrain might contribute to trigger dopamine neurodegeneration in PD. Indeed, systemic administration of LPS (5 mg/kg) caused long-term dopaminergic degeneration and microglial activation in mice [25]. Therefore, another interpretation of the results obtained in this study would be that the early immunosuppressive response to LPS elicited by the midbrain might contribute to dopaminergic neuronal death rather than prevent cell damage. In fact, Treg depletion and blockade of the PD-1/PD-L1 immune checkpoint pathway improved cognitive performance and amyloid-β pathology in experimental models of Alzheimer’s disease [54–56]. Growing body of evidence shows that peripheral adaptive immunity is involved in PD. PD patients present dysfunctional Treg cells that might contribute to a persistent inflammatory environment or to a loss of tolerance to α-synuclein [57–59]. Since the BBB is compromised in PD [60], it could be hypothesized that these lymphocytes could infiltrate the brain and interact with microglial cells. We hypothesize that inflammation plays a dual role in neuronal degeneration. An exacerbated immunosuppressive response such as the one that we describe in the midbrain after LPS administration or an excessive pro-inflammatory effect might counterbalance the beneficial effect of an equilibrated inflammatory tone and would compromise survival of dopaminergic neurons. Additional experiments are needed in experimental models of PD to dissect out the pathogenic and protective roles of inflammation in PD.

## Conclusion

Our data show that under steady-state conditions, the neuroinflammatory tone of the brain differs from one region to another. In this regard, the midbrain has unique properties, being the region that presents the highest immune-alert state and unique microglial subpopulations. These differences have an impact in the type of inflammatory response since a specific immune-suppressive effect after systemic LPS administration was identified in the midbrain, but not in other brain regions. Further studies will be necessary to determine how the populations of midbrain microglia described in this study are activated by different stimuli, or in experimental models of PD, and to assess their presence in the human brain.

## Supplementary information

**Additional file 1:** **Figure S1.** Control of signal specificity in the cell surface analysis of pro-inflammatory markers. **Figure S2.** Gating of purified cells from adult mice brain for flow cytometry analysis. **Figure S3.** Unique and non-overlapping subpopulations of microglial cells in the midbrain. **Figure S4.** Positive control of CD4+ T cell proliferation after presentation of the OVA peptide by CD11c+splenocytes. **Table S1.** List of overlapping genes differentially expressed in the striatum compared to midbrain in the two experiments

## Data Availability

RNA sequencing data used to generate Fig. 5 are publicly available in the GEO database with the accession number GSE133617. The data that support the findings of the rest of the study are available from the corresponding author upon reasonable request.

## Abbreviations

APC: Antigen-presenting cell
BBB: Blood-brain barrier
CNS: Central nervous system
CTLA-4: Cytotoxic T lymphocyte antigen 4
FACS: Fluorescence-activated cell sorting
FSC: Forward scatter
LPS: Lipopolysaccharide
MFI: Median fluorescence intensity
MHC-I: Major histocompatibility complex class I
MHC-II: Major histocompatibility complex class II
PD: Parkinson’s disease
PD-L1: Programmed death ligand 1
SN: Substantia nigra
SNc: SN pars compacta
SNr: SN pars reticulata
SSC: Side scatter
TLR4: Toll-like receptor 4
VTA: Ventral tegmental area
