# Supplementary material for: Midbrain microglia mediate a specific immunosuppressive response under inflammatory conditions
Source: J Neuroinflammation. 2019 Nov 22;16:233. doi: 10.1186/s12974-019-1628-8 (PMC6874825; doi:10.1186/s12974-019-1628-8)
Supplement: Supplementary file 1 — Additional file 1: Figure S1. Control of signal specificity in the cell surface analysis of pro-inflammatory markers. Figure S2. Gating of purified cells from adult mice brain for flow cytometry analysis. Figure S3. Unique and non-overlapping subpopulations of microglial cells in the midbrain. Figure S4. Positive control of CD4+ T cell proliferation after presentation of the OVA peptide by CD11c+splenocytes. Table S1. List of overlapping genes differentially expressed in the striatum compared to midbrain in the two experiments [file 12974_2019_1628_MOESM1_ESM.docx]

**Supplementary Material**

**
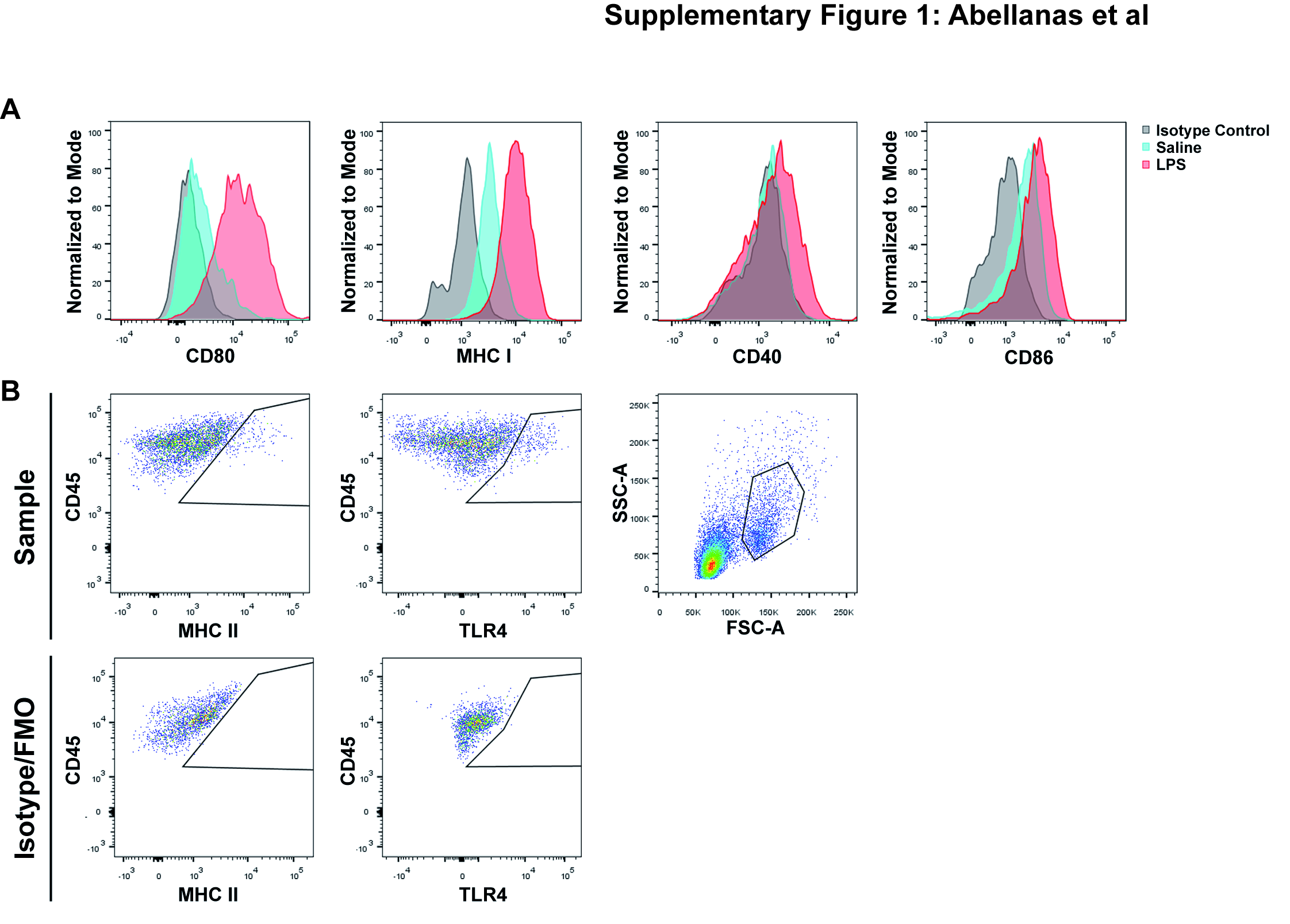
**

**Supplementary Figure 1.** Control of signal specificity in the cell surface analysis of pro-inflammatory markers. (A) Microglial cells (CD45^low^/CD11b^+^) were marked with an isotype antibody for CD80 or fluorescence minus one (FMO) controls for MHC-I, CD40 and CD86. Isotype and FMO controls are represented in grey, microglial cells from control mice in blue and microglia from LPS-treated mice in red. (B) Gating strategy to analyze microglial (CD45^low^/CD11b^+^) subpopulations expressing MHC-II, TLR4 or FSC^hi^/SSC^hi^. Cells were marked with the isotype control antibody for MHC-II and a FMO control was performed for TLR4. Representative dot-plots are shown.


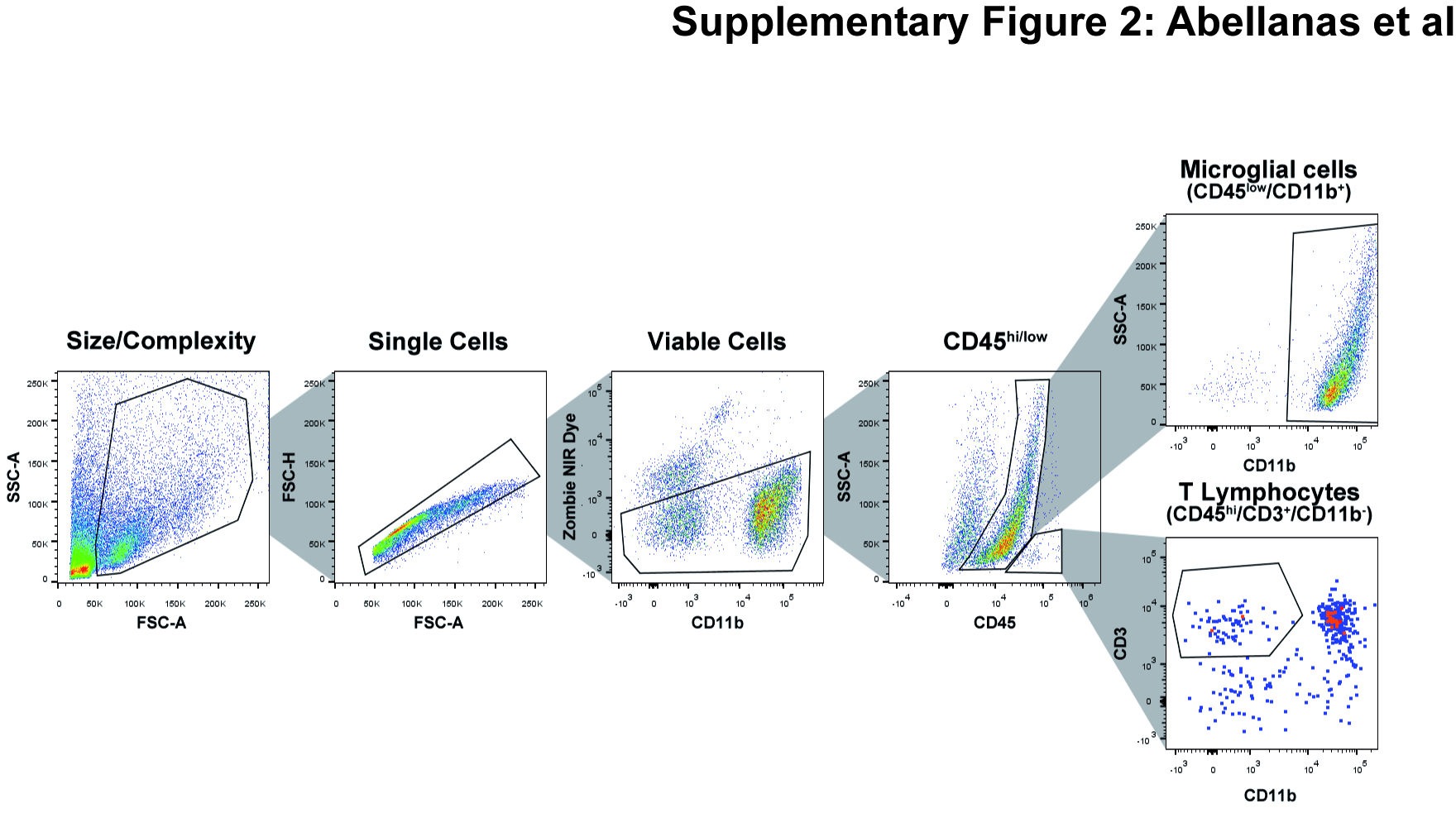


**Supplementary Figure 2.** Gating of purified cells from adult mice brain for flow cytometry analysis. From the total events recorded, subpopulations of interest were selected based on the forward and side scatter (FSC and SSC). Single cells were determined as the events that were linearly proportional in the area and height of the forward scatter signal (FSC-A and FSC-H), and viable cells were identified as cells negative for the Zombie NIR Viability Dye (BioLegend). Immune cells were identified by their CD45 expression, and microglia had weak CD45 expression and they were positive for CD11b (CD45^low^/CD11b^+^). T-cells were characterized as the CD45^hi^/CD11b^-^/CD3^+^ population.


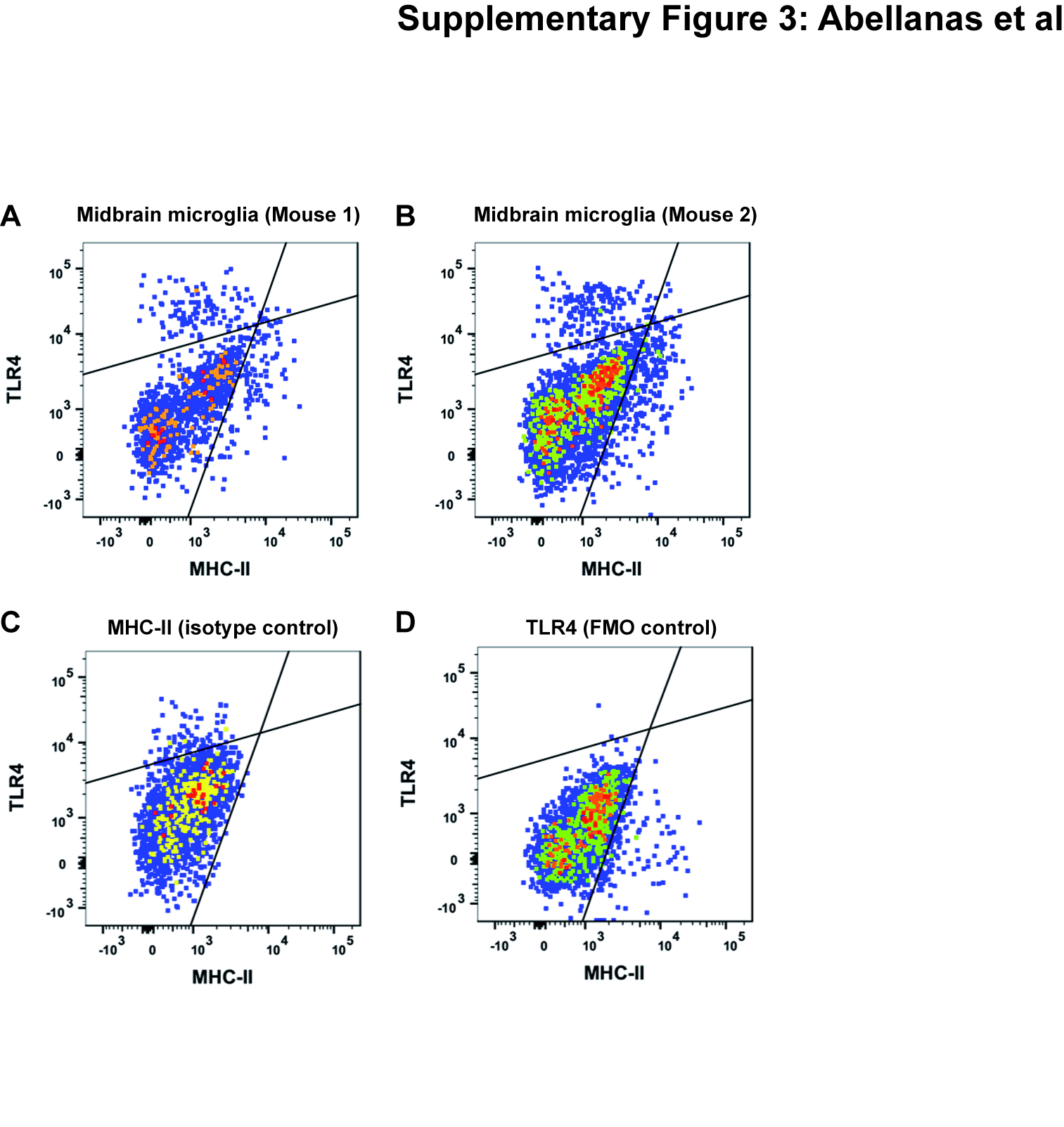


**Supplementary Figure 3:** Unique and non-overlapping subpopulations of microglial cells in the midbrain. Gating strategy for MHC-II^+^ and TLR4^+^ microglial cells, (A and B) show representative dot plots of midbrain microglial cells from two control animals, MHC-II^+^/TLR4^+^ cells are almost undetectable. (C) Dot plot of microglial cells incubated with isotype control of MHC-II antibody. (D) Dot plot of microglial cells marked with a FMO mix without anti-TLR4 antibody.

**
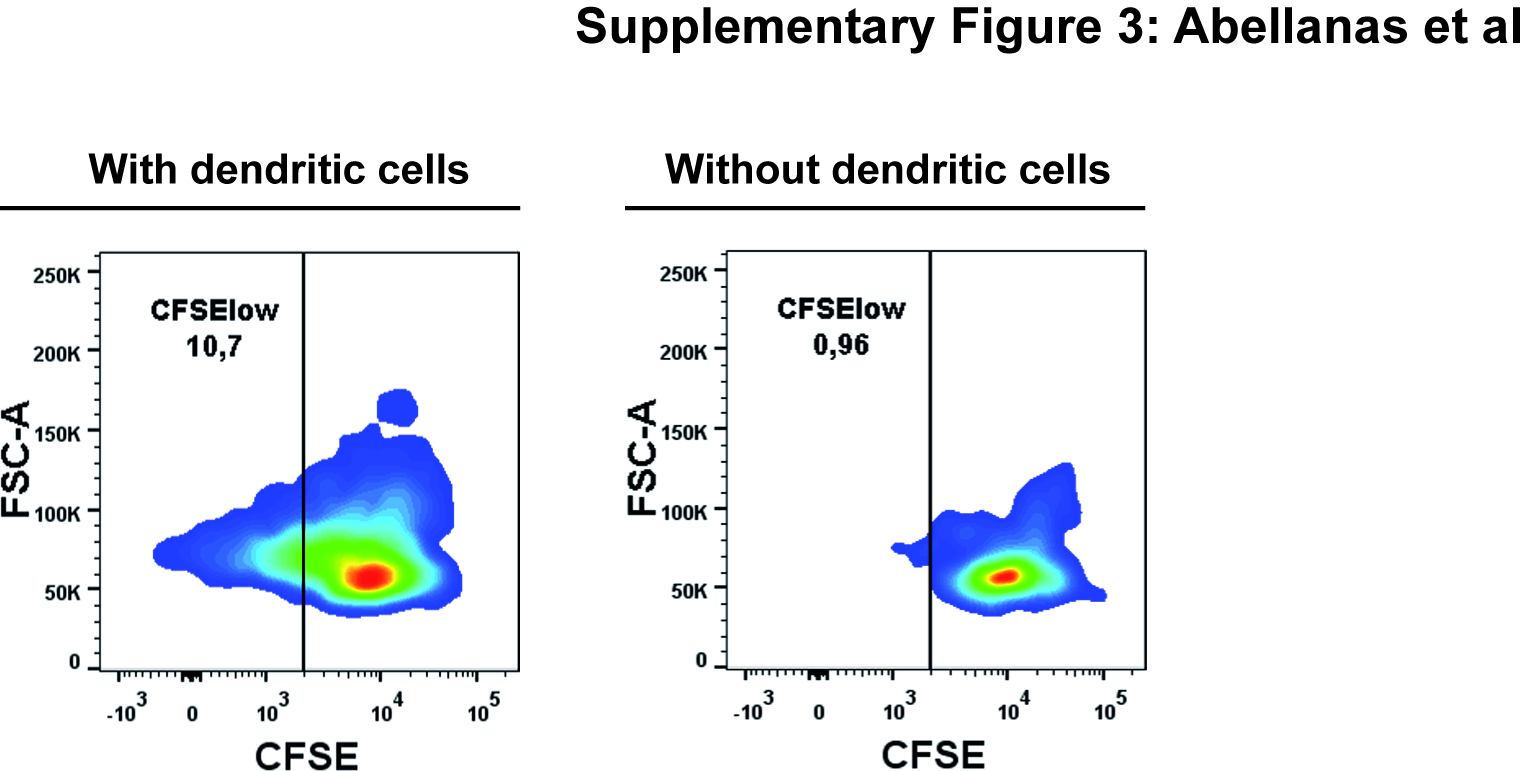
**

**Supplementary Figure 3.** Positive control of CD4^+^ T cell proliferation after presentation of the OVA peptide by CD11c^+^splenocytes. Flow cytometry analysis of CFSE-stained CD4^+^ T cells after 7 days in co-culture with CD11c^+^ dendritic cells obtained from the spleen, or without APCs. Representative density plots selected from one experiment of three: CFSE, carboxyfluorescein succinimidyl ester; FSC, forward scatter.
